# Life satisfaction of Palestinian and Polish students after pandemic COVID-19

**DOI:** 10.3389/fpubh.2024.1409710

**Published:** 2025-01-29

**Authors:** Krzysztof Zdziarski, Anna Knyszyńska, Katarzyna Karakiewicz-Krawczyk, Mariam Awad, Salam Awad, Narmeen Qumsieh, Marek Landowski, Beata Karakiewicz

**Affiliations:** ^1^Subdepartment of Social Medicine and Public Health, Department of Social Medicine, Pomeranian Medical University, Szczecin, Poland; ^2^Faculty of Nursing and Health Sciences, Bethlehem University, Bethlehem, Palestine; ^3^Independent Research and Biostatistics Laboratory, Department of Social Medicine, Pomeranian Medical University, Szczecin, Poland

**Keywords:** sense of happiness, life satisfaction, student, COVID-19 pandemic, Palestine, Poland

## Abstract

The COVID-19 pandemic has had a huge impact on the lives of all social groups around the world, including students who have had to face remote learning and isolation. Regardless of cultural, linguistic and religious differences, every young person is looking for the meaning of life and their place in the world. This process includes physical, mental, social and spiritual aspects. The pandemic has highlighted the importance of these elements anew, especially in the face of threats to health and life. The study included two groups of students: 238 from Palestine and 293 from Poland. The question was asked: what factors have the greatest impact on students’ sense of happiness after the COVID-19 pandemic? The results showed interesting differences in their approach to life and happiness. Palestinian students perceive life more optimistically and attach importance to family ties, physical health and religion. Polish students attach importance to social activity, meetings with friends, traveling, listening to music and watching movies. For them, these forms of spending free time are important in the context of mental regeneration and escape from stress. They emphasize that a good financial situation gives them a sense of security and allows them to pursue their passions and interests. Both groups do not associate happiness with psychoactive substances. The results indicate a generally positive assessment of students’ life satisfaction after the pandemic. Research shows that there are universal values that give a sense of happiness and life satisfaction to young people around the world.

## 1 Introduction

The COVID-19 pandemic has caused disruptions in the existence of human populations around the world. Scientific research reports emphasize that the pandemic harmed health in all its dimensions (1). The everyday life of academic youth was disturbed by external factors, which also caused emotional and mental disorders. It was noticed, among other things, that participation in social life was impaired, and stress caused high levels of anxiety, depression and social phobias (2, 3). Research shows that the social mentality of students has also decreased (4). The time after the pandemic has become an opportunity to reflect on the effectiveness of administered vaccines and the value of life from the perspective of the past. This perspective includes the philosophical understanding of existential space, which becomes a place of interaction between man and the world and other people. The way man experiences this space depends on his actions and living conditions (5, 6).

It should be noted here that during the pandemic, the term existential, which from ex-sistere means to become, to emerge, as a result of a threat to life, contact with death, thinking about death, has taken on the meaning of existential anxiety (7, 8). Scientists emphasize that existential anxiety negatively affects human life and can cause anxiety disorders and depressive mood disorders (9, 10). However, it can also have positive effects, because experiencing it can promote the activation of prosocial motivation, empathy, tolerance, forgiveness. It can also contribute to increased engagement in pro-health and helping behaviors and promote greater innovation, creativity, and flexibility in action (11, 12).

The existential spaces selected for research were Palestinian and Polish as 2022 research on these same populations showed that the administered vaccines against COVID-19 contributed to the strengthening of vital forces and gave a sense of security in a person’s everyday functioning (13). Respondents showed that accepting the vaccines was their free choice, and the social motivation was active participation in public life and the ability to travel freely in the case of Polish respondents, and in the case of Palestinian respondents – fear of infecting others. From an existential, psychosocial, and health perspective, vaccines have had a comparatively significant impact on young people’s social well-being in Palestine and Poland (14–17).

The research conducted from June to September 2023 covers two social groups. The first one was from the Palestinian area, in health-threatening conditions, and the Polish respondent, living in a safe existential space. It should be emphasized that the research was conducted before the start of the Israeli-Palestinian war (7/10/2023), and the Palestinian respondents had been witnesses or participants in the Israeli-Palestinian conflict for many years.

Hence, their existential situation was defined as health-threatening. Taking into account these experiences, it can be assumed that they were more resistant to overcoming the difficulties of the COVID-19 pandemic. The health threat to Palestine resulting from the long-term armed conflict falls within the definition of an existential threat, which has many meanings and indicates, among others, a threat to survival and loss of one’s own identity (18). Hence, hypothetically, it can be assumed that their life satisfaction after the pandemic should be higher than that of Polish students who did not experience difficulties related to international conflicts.

It is worth emphasizing that scientific reports indicate large fluctuations in the uptake of vaccinations in conflict areas, which largely also applies to Palestinian respondents (19), and the lack of availability of vaccines, which differentiates these two populations (20). In this perspective, the life satisfaction is influenced by all spheres of everyday existence: physical, mental, emotional, social, material and spiritual. It is crucial to look at these spheres from the perspective of a participant, often traumatic experiences brought about by the pandemic. Experiencing difficulties and overcoming them generates a new life perspective and axiology, which sets a new existential path to personal well-being (21–25). Psychological research indicates that assessing oneself and others in the context of difficult life events (pandemics) is a potential source of acquired resilience in young people (26). As scientists emphasize, people with a high level of ego resilience show a higher level of life satisfaction (27).

The lifestyle of young people that has developed after the COVID-19 pandemic, according to scientific research, is characterized by greater concern for one’s own life, conformism, self-direction and less hedonism. People who were more open to changes before the pandemic show higher eudaimonism after the pandemic (28). Positive emotions are the most frequently indicated factor by students influencing increased resilience and satisfaction with life. Scientific research confirms that people become more satisfied when they develop resources for a good life and feel good. And good well-being is associated with personality factors: extraversion, optimism and a general sense of personal competence (29, 30).

The aim of the research conducted at universities in Palestine and Poland was to indicate what factors and to what extent they affect students’ life satisfaction after the COVID-19 pandemic. Students’ opinions combined with research results showing the inconveniences of living during a pandemic are an impulse for a broader discussion on methods that generate safety for the human population in conditions of health risk. Moreover, they provide an opportunity to conduct a discourse on the effectiveness of measures used to protect life and health. Palestine and Poland are countries with different cultural, social and political contexts. Conducting comparative research between these two groups of students allows us to understand how these differences have influenced perceptions of life satisfaction after the pandemic. Cultural norms and social structures differentiate the way young people cope with, among other things, stress and changes caused by the pandemic. Palestinian students may experience additional difficulties related to political conflict and restrictions in everyday life, which have a significant impact on their life satisfaction.

In Poland, the pandemic has affected students in ways that are more related to economic, educational and social issues. The studies conducted so far significant information about the course and negative effects of the COVID-19 pandemic. Publications cover issues related to depression, anxiety, stress (31–33), negative effects of distance learning (34), knowledge about the pandemic and good preventive practices among young people (35). The diverse impact of COVID-19 on Arab countries (36), the impact of social support on coping strategies in difficult life situations and on the quality of life of teenagers (37) were examined. Extensive studies were also conducted on the life satisfaction of students at nine universities during the first wave of the COVID-19 pandemic (38). It was also checked to what extent psychoactive substances are used to overcome psychoactive difficulties during the pandemic (39). The impact of health-promoting behaviors on life satisfaction and a protective factor against stress during the third wave of the SARS-CoV-2 pandemic was examined (40).

The impact of meaning in life, life satisfaction, and beliefs about order and positivity of the social world on emotional and cognitive reactions to the pandemic was measured (41). Also, in the largest cities in Poland, the level of fear of the pandemic, life satisfaction, and mental health were measured in the initial phase of the pandemic (42). In this perspective, taking into account the problem of students’ life satisfaction after the COVID-19 pandemic, it should be noted that there are already scientific reports in this area, including those on the quality of life of students before and during the pandemic (43, 44), the impact of the Internet on life satisfaction before and after the pandemic, as well as the impact of online classes on well-being (45). Research has also been conducted on interpersonal communication using dance and movement therapy (46) and potential challenges in the area of improving mental health and regaining life satisfaction after the pandemic (47).

The presented research results indicate factors that affect the life satisfaction of students from two cultures who lived in different psychosocial conditions during the pandemic. Their direct, existential experience of the pandemic gave the opportunity to reveal subjective feelings after its end. Scientific interpretations of the pandemic strongly influence two aspects of human experience: the human’s embeddedness and connection with the material reality in which they live, and the freedom to act in any chosen way. Problems related to social isolation, adversity, depression, anxiety, and emotional difficulties are closely connected with existential experience (48).

The study also provides information on how students in both countries may perceive their future after the pandemic, which is a key element of their overall life satisfaction. Understanding these differences can help develop programs that are more tailored to the specific needs of young people in different cultural and geographical contexts. By comparing the lives of students from both countries, we can better understand how political stability or lack thereof affects education, access to resources, mental health and overall life satisfaction. Comparing Polish and Palestinian students shows how the pandemic has further deepened existing inequalities, as well as which factors of coping with the pandemic have had a positive impact on everyday life. Students in both countries need psychological support, but the reasons and scope of this support differ significantly. Palestinian students struggle with war trauma, while Polish students feel more stressed due to academic pressure and uncertainty in the job market. In both countries, young people strive for personal development, but their opportunities and the obstacles they encounter differ (49).

Comparing the experiences of both populations can help identify universal youth needs and specific challenges resulting from different socio-political contexts, and show how different cultures and communities influence young people’s educational and life decisions. Finally, comparing the situation in two different countries can lead to the identification of good practices in educational policy that can be adapted or inspire changes in other countries. Comparing life satisfaction among Palestinian and Polish students after the COVID-19 pandemic can provide valuable information on the impact of global crises on the young generation in different parts of the world. It also allows for a better understanding of the impact of the social, political and economic context on the education and lives of young people. This allows for drawing conclusions that can serve both researchers and policymakers as well as organizations that support young people (50).

## 2 Methods

### 2.1 Study design and settings

The research was designed and carried out in Palestine and Poland as part of the Erasmus+ program implemented in Bethlehem by the research and teaching staff of the Pomeranian Medical University. Data collection started in June 2023 and ended at the end of September 2023. Both universities have been cooperating since 2018 and previously conducted research projects on attitudes toward death and subjective feelings after receiving vaccinations against COVID-19.

### 2.2 The questionnaire

The study used a survey questionnaire which consisted of 35 questions including sociodemographic and detailed questions using the Satisfaction With Life Scale (SWLS) from the Department of Psychology of the University of Illinois. Life satisfaction assessed on the SWLS scale is expressed in the sense of satisfaction with one’s achievements and conditions. The main features of SWLS are its simplicity and universality. The scale can be used in various populations, regardless of age, culture or social status, which makes it a versatile tool in research on human well-being. During the COVID-19 pandemic, this tool is most often used by researchers, because it allows for a holistic assessment, giving the possibility of independent interpretation of which aspects of life affect overall satisfaction in conditions of health risk (51–56).

Construct validity is verified by analyzing criteria that indirectly reflect the sense of life satisfaction. The Likert scale was used for detailed statements included in the survey, where respondents could choose one of three answers: 1 – I disagree; 2 – neither agree nor disagree; 3 – I agree. The choice of a 3-point Likert scale in this study was preceded by a review of other studies and scientific articles that also used this scale. For example, in the work (57) the pregnancy-specific stress are rated in 3-point Likert scale. Questions regarding attitudes toward COVID-19 preventive measures and hygiene recommendations in the article (58) are rated in 3-point scale. The attitude toward the COVID-19 infection among adults in the paper (59) are rated in 3-point Likert scale. In the article (60), the authors used a 3-point Likert scale to measure preventive behaviors toward COVID-19 infection, from never to often/always. In many scientific articles, the authors use a 3-point Likert scale. The link to the survey was sent by the researchers to students from both universities. Before starting the study, respondents were informed that by completing the survey they agreed to use their anonymous data for scientific purposes. Online respondents completed the survey anonymously at Palestinian and Polish universities and could stop the research at any time.

The first part of the survey included sociodemographic questions (gender, age, field and year of study, place of residence, marital status), and the second part included detailed statements (I take care of my physical condition – I practice sports, eat healthy food, drink alcohol, smoke traditional cigarettes, smoke cigarettes electronic, I use psychoactive substances, I meet friends, I have time for friends, I go to youth clubs, I travel, I go on trips, I can relax, I read a lot, listen to music, watch movies, I spend a lot of time on the phone, computer, laptop, I gamble, I have good relationships at home, I am guided by spiritual/religious values in my life, I practice my religion, I support myself with the help of my parents, I work gainfully, I assess my financial situation well, I am depressed, I feel loneliness, in many respects my life is similar, it’s perfect, my living conditions are perfect, I’m satisfied with my life, I’ve achieved the most important things in my life that I wanted, if I could live my life over again, I would not want to change almost anything).

### 2.3 Study participants and sample size

Five hundred and thirty-one students took part in the study, 293 from Poland and 238 from Palestine. The response rate is 100%. Of the group of Polish students, 84% were women, and from Palestine, 81%. The average age of respondents from Poland and Palestine is 20.22 and 20.43 years. Considering the age of respondents by gender, women from Poland and Palestine have an average age of 20.09 and 20.51 years, and men are 20.94 and 20.11 years old. Most students from Poland are students of the following fields: psychology 25%, speech therapy 18%, cosmetology 16%, physiotherapy 14%, medicine 9%, medical analytics 5%. Among students from Palestine, the largest group of respondents were students in the following fields: nursing 80%, midwifery 9%, occupational therapy 3%, and physiotherapy 3%.

Considering the place of residence of the respondents, it should be noted that from the group of Polish students, 60% of people live in a large city (over 100,000 inhabitants), 26% in a small town (up to 100,000 inhabitants), and the remaining 14% of Polish respondents live in the countryside. Among Palestinian respondents, 32% live in a large city and the remaining 68% live in a small town. 46% of respondents from Poland and 15% from Palestine live in a civil partnership, the remaining respondents are not in a relationship with anyone. Table 1 quantifies the characteristics and the mean and standard deviation of the age of people taking part in the survey concerning nationality, gender and age range.

**Table 1:** Number of respondents and means and standard deviations of their age concerning nationality, gender and age ranges.

Group of respondents	Polish students		Palestinian students	
	*N*	Age (Mean ± SD)	*N*	Age (Mean ± SD)
All respondents	293	20.22 ± 2.38	238	20.43 ± 3.25
Gender				
Women	246	20.09 ± 2.43	192	20.51 ± 3.52
Men	47	20.94 ± 1.85	46	20.11 ± 1.77
Age				
<20 years old	130	18.6 ± 0.55	88	18.43 ± 0.56
20–23 years old	143	20.92 ± 0.96	142	21.06 ± 0.87
>23 years old	20	25.75 ± 4.83	8	31.38 ± 11.99

### 2.4 Data analysis

The research used a three-grade Likert scale. To those presented in the statement survey, the respondent had the opportunity to choose one of three answers: 1 – I do not agree; 2 – neither agree nor disagree; 3 – I agree. Cronbach’s alpha coefficient for questions about Polish and Palestinian students is 0.77 and 0.86, respectively, so the overall reliability of the questionnaire was achieved. Quantitative data were presented as mean and standard deviation, as well as percentages and graphically using histograms. The chi-squared test was used to analyze differences between distributions. A significance level of 0.05 was adopted for all tests. Cronbach’s alpha was used to measure the reliability of questions with answers given on a Likert scale.

## 3 Results

Respondents were asked to respond to statement S1 (“I take care of my physical condition”), 69.7% of students from Palestine and 62.1% of students from Poland confirm this statement. By performing a chi-square test, it was shown that the response to the statement depended on the respondents’ nationality (*p* < 0.05). The result of the chi-square test shows that nationality has no influence on the answer to question S2 (“I eat healthy food”). 64.7 and 63.1% of students from Palestine and Poland, respectively, confirmed facts about nutrition.

Similarly, using the chi-square test, no statistically significant relationships were found between the nationality of respondents and answers to questions S3 (“I smoke traditional cigarettes”) (*p* > 0.05) and S4 (“I use psychoactive substances”), *p* > 0.05. Regarding questions about smoking, 90.3 and 92.2% of respondents from Palestine and Poland, respectively, said they did not smoke cigarettes. A higher rate of 92 and 95.6% among Palestinian and Polish students, respectively said that students were drug-free. However, 5% of the surveyed Palestinians and 2.4% of Poles use psychoactive substances. Questions S5 (“I meet my friends”) is answered analogously, with a chi-square test that has no additional application and is statistically dependent. About 80% of respondents meet with friends. To obtain an answer to the question S6 (“I have time for friends”), a chi-squared test was determined by the student’s nationality (*p* < 0.05). More students from Poland declared that they had time for friends than students from Palestine. The percentage results correspond to 80.9 and 64.3%. A large group of students from Palestine (24.8%) along with students from Poland (8.9%) stated that they had no time for friends.

Considering questions S7 (“I go to youth clubs”), S8 (“I travel, I go on trips”), S9 (“I can relax”), S11 (“I listen to music”), S12 (“I watch movies”) and S13 (“I spend a lot of time on the phone”), in all these cases the chi-square test showed that the answers depended on the respondents’ country of origin (*p* < 0.05). For example, 38.6% of respondents from Poland attend youth clubs, compared to 14.3% for respondents from Palestine. 66.6% of Polish students and 35.7% of Palestinian students travel. 73.4% of Polish students and 60.5% of Palestinian students relax. Listen to music (S11) and watching movies (S12) were 94.2 and 86.7% of Polish students, respectively, and 70.2 and 64.7% of Palestinian students, respectively. Spending a lot of time using the phone confirmed – 86.3% of Polish students and 69.3% of Palestinian students.

Conducting a chi-square test based on question asked to respondents whether they read a lot (statement S10), the answers of Polish and Palestinian students were found not to be significantly different (*p* > 0.5). The fact that 54.3 and 51.3% of students from Poland and Palestine read a lot (statement S10), respectively. 30.4 and 34.9% of respondents do not agree with this statement.

Statistically significant differences between the responses of students from Palestine and Poland were detected using the chi-square test in responses to statements: S14 (“I have good relationships at home”), S15 (“In my life I am guided by spiritual/religious values”) and S16 (“I practice my religion”). There were more positive responses among the students from Palestine. 85.3% of respondents from Palestine and 73.7% from Poland agreed with the statement S14. Statement S15 was confirmed by 85.3% of Palestinian students and 26.3% of Polish students. A similar situation occurred in responses to the statement S16. 87.8% of students from Palestine and 21.8% from Poland agree with this statement.

The survey also asked respondents about their financial affairs. The chi-square test showed that the answers provided depended on the respondents’ country of origin (*p* < 0.05) for the statements S17 (“I support myself with the help of my parents”), S18 (“I am gainfully employed”) and S19 (“I assess my financial situation well”). It can be concluded that fewer Polish students benefit from financial support from their parents. 87.8% of Palestinian students and 78.2% of Polish students agreed with the statement: I support myself with the help of my parents. Paid work was confirmed by 44.7% of Polish respondents and 31.5% of Palestinian respondents.

However, 77.5% of students from Poland and 53.8% from Palestine rated their financial situation as good. For the next statement S20 (“I have depression”), the survey confirmed a statistically significant relationship between the answers provided and the nationality of the respondents using the chi-square test (*p* < 0.05). Among students from Poland, 35.8% indicated that they felt depressed, among students from Palestine this percentage was 23.5%. However, 50.9% of students from Poland and 63% from Palestine disagree with this statement.

The next statement presented to respondents was S21 (“I feel lonely”). The chi-square test showed that the answers to this question depended on the nationality of the respondents (*p* < 0.05). The percentage of respondents from Poland who disagree with this statement is 46.4%, while from Palestine – 66.8%. However, 42.4% of Palestinian students and 29% of Polish students believe that “In many ways my life is close to ideal” (statement S22), while 39.9 and 44% are of the opposite opinion. Also in the case of this question, the chi-square test showed that the answers depended on the respondents’ nationality (*p* < 0.05).

To the statement S23 (“My living conditions are perfect”), 51.3% of Palestinians and 46.4% of Polish students responded affirmatively, while 29.8 and 30.4%, respectively, denied it. The chi-square test showed that the answers to this question did not depend significantly on the nationality of the respondents (*p* > 0.05).

In question S24 (“I am satisfied with my life”), 74.4% of Palestinians and 62.8% of Poles confirm that they are satisfied with their lives. 16.8 and 18.1%, respectively, deny this statement. The result of the chi-square test shows that the answer statistically significantly depends on the respondent’s country of origin. Despite the young age of most respondents, 54.2% of Palestinian students and 31.1% of Polish students confirm that they achieved what they wanted most in life, S25 (“I achieved the most important things in life that I wanted”). However, 47.1% of Poles and 27.7% of Palestinians have the opposite opinion. In this case, the chi-square test indicates a statistically significant dependence of the answers on the nationality of the surveyed students.

**Figure 1:**
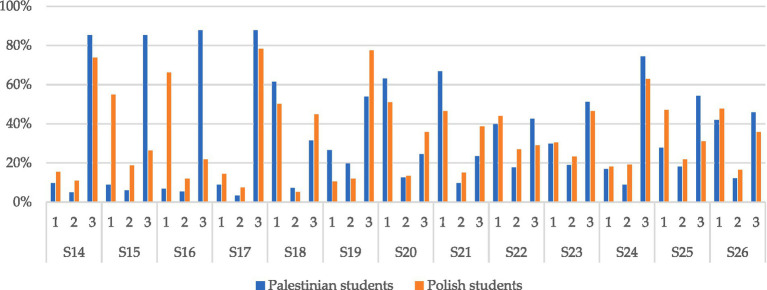
Percentage of answers to individual questions based on respondent nationality.

The last question S26 in the survey is: “If I could live my life over again, I would not want to change almost nothing.” The responses show that 45.9% of Palestinian students and 35.8% of Polish students agree with this statement. 42% of students from Palestine and 47.8% from Poland disagree with this statement. The result of the chi-square test indicates that the answers do not depend on the nationality of the respondents. To assess the effect size of the statistically significant association, the Cramer’s *V* value was also calculated. The interpretation of effect size (ES) was made according to the scale provided on the IBM website^1^, i.e., strong for ES > 0.6, moderate 0.2 < ES ≤ 0.6, weak for ES ≤ 0.2. Strong association was obtained for statement S15, moderate for S6, S7, S8, S11, S12, S13, S15, S19, S21, S25, weak for S1, S9, S14, S17, S18, S20, S22, and S24. Detailed data of the results described above are presented in Table 2, and graphical percentages are presented in Figure 1.

**Table 2:** Comparison of answers to individual questions based on respondent nationality.

No	Statement	Student’s nationality	Answer *N* (%)			Chi-Squared Test		
			1	2	3	Chi-Sq	Df	*p*
S1.	I take care of my physical condition	Palestinian	50 (21%)	22 (9.2%)	166 (69.7%)	6.6636	2	0.0357
		Polish	62 (21.2%)	49 (16.7%)	182 (62.1%)			
S2.	I eat healthy food	Palestinian	46 (19.3%)	38 (16.0%)	154 (64.7%)	0.9625	2	0.6180
		Polish	52 (17.7%)	56 (19.1%)	185 (63.1%)			
S3.	I smoke traditional cigarettes	Palestinian	215 (90.3%)	7 (2.9%)	16 (6.7%)	0.6546	2	0.7209
		Polish	270 (92.2%)	6 (2%)	17 (5.8%)			
S4.	I use psychoactive substances	Palestinian	219 (92%)	7 (2.9%)	12 (5%)	3.1870	2	0.2032
		Polish	280 (95.6%)	6 (2%)	7 (2.4%)			
S5.	I meet my friends	Palestinian	27 (11.3%)	23 (9.7%)	188 (79%)	3.4841	2	0.1752
		Polish	26 (8.9%)	18 (6.1%)	249 (85%)			
S6.	I have time for friends	Palestinian	59 (24.8%)	26 (10.9%)	153 (64.3%)	22.8872	2	<0.0001
		Polish	29 (9.9%)	27 (9.2%)	237 (80.9%)			
S7.	I go to youth clubs	Palestinian	190 (79.8%)	14 (5.9%)	34 (14.3%)	41.9291	2	<0.0001
		Polish	158 (53.9%)	22 (7.5%)	113 (38.6%)			
S8.	I travel, I go on trips	Palestinian	124 (52.1%)	29 (12.2%)	85 (35.7%)	63.8906	2	<0.0001
		Polish	58 (19.8%)	40 (13.7%)	195 (66.6%)			
S9.	I can relax	Palestinian	56 (23.5%)	38 (16%)	144 (60.5%)	10.0089	2	0.0067
		Polish	48 (16.4%)	30 (10.2%)	215 (73.4%)			
S10.	I read a lot	Palestinian	83 (34.9%)	33 (13.9%)	122 (51.3%)	1.2439	2	0.5369
		Polish	89 (30.4%)	45 (15.4%)	159 (54.3%)			
S11.	I listen to music	Palestinian	50 (21%)	21 (8.8%)	167 (70.2%)	54.9802	2	<0.0001
		Polish	11 (3.8%)	6 (2%)	276 (94.2%)			
S12.	I watch movies	Palestinian	63 (26.5%)	21 (8.8%)	154 (64.7%)	42.9075	2	<0.0001
		Polish	19 (6.5%)	20 (6.8%)	254 (86.7%)			
S13.	I spend a lot of time on the phone	Palestinian	50 (21%)	23 (9.7%)	165 (69.3%)	26.1768	2	<0.0001
		Polish	20 (6.8%)	20 (6.8%)	253 (86.3%)			
S14.	I have good relationships at home	Palestinian	23 (9.7%)	12 (5%)	203 (85.3%)	11.0335	2	0.004
		Polish	45 (15.4%)	32 (10.9%)	216 (73.7%)			
S15.	In my life I am guided by spiritual/religious values	Palestinian	21 (8.8%)	14 (5.9%)	203 (85.3%)	185.043	2	<0.0001
		Polish	161 (54.9%)	55 (18.8%)	77 (26.3%)			
S16.	I practice my religion	Palestinian	16 (6.7%)	13 (5.5%)	209 (87.8%)	234.796	2	<0.0001
		Polish	194 (66.2%)	35 (11.9%)	64 (21.8%)			
S17.	I support myself with the help of my parents	Palestinian	21 (8.8%)	8 (3.4%)	209 (87.8%)	8.8447	2	0.012
		Polish	42 (14.3%)	22 (7.5%)	229 (78.2%)			
S18.	I am gainfully employed	Palestinian	146 (61.3%)	17 (7.1%)	75 (31.5%)	9.7596	2	0.0076
		Polish	147 (50.2%)	15 (5.1%)	131 (44.7%)			
S19.	I assess my financial situation well	Palestinian	63 (26.5%)	47 (19.7%)	128 (53.8%)	34.9362	2	<0.0001
		Polish	31 (10.6%)	35 (11.9%)	227 (77.5%)			
S20.	I have depression	Palestinian	150 (63%)	30 (12.6%)	58 (24.4%)	9.1306	2	0.0104
		Polish	149 (50.9%)	39 (13.3%)	105 (35.8%)			
S21.	I feel lonely	Palestinian	159 (66.8%)	23 (9.7%)	56 (23.5%)	22.1409	2	<0.0001
		Polish	136 (46.4%)	44 (15%)	113 (38.6%)			
S22.	In many ways, my life is close to ideal	Palestinian	95 (39.9%)	42 (17.6%)	101 (42.4%)	12.2861	2	0.0021
		Polish	129 (44%)	79 (27%)	85 (29%)			
S23.	My living conditions are perfect	Palestinian	71 (29.8%)	45 (18.9%)	122 (51.3%)	1.7885	2	0.4089
		Polish	89 (30.4%)	68 (23.2%)	136 (46.4%)			
S24.	I am satisfied with my life	Palestinian	40 (16.8%)	21 (8.8%)	177 (74.4%)	12.2972	2	0.0021
		Polish	53 (18.1%)	56 (19.1%)	184 (62.8%)			
S25.	I achieved the most important things in life that I wanted	Palestinian	66 (27.7%)	43 (18.1%)	129 (54.2%)	30.7298	2	<0.0001
		Polish	138 (47.1%)	64 (21.8%)	91 (31.1%)			
S26.	If I could live my life over again, I would not want to change almost anything	Palestinian	100 (42%)	29 (12.2%)	109 (45.8%)	5.7951	2	0.0552
		Polish	140 (47.8%)	48 (16.4%)	105 (35.8%)			

Answer: 1 – I do not agree, 2 – I neither agree nor disagree, 3 – I agree.

**Figure 2:**
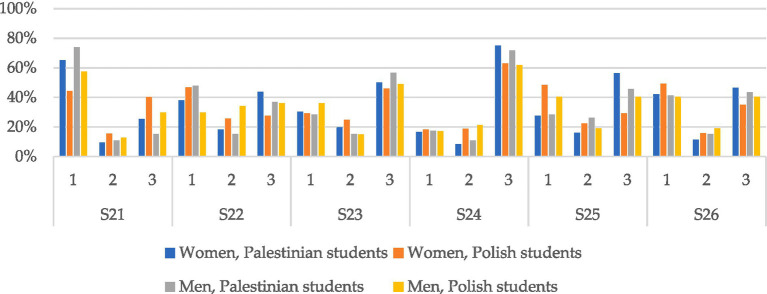
Percentage of three types of answers to individual questions based on respondent nationality and gender.

Additionally, the answers to selected survey questions were examined according to the respondents’ gender, the results are presented in Table 3 and graphically in Figure 2.

**Table 3:** Comparison of three types of answers to selected individual questions based on respondent nationality and gender.

No	Statement	Gender	Student’s nationality	Answer *N* (%)			Chi-Square Test		
				1	2	3	Chi-Sq	Df	*p*
S21.	I feel lonely	Women	Palestinian	125 (65.1%)	18 (9.4%)	49 (25.5%)	18.7563	2	<0.0001
			Polish	109 (44.3%)	38 (15.4%)	99 (40.2%)			
		Men	Palestinian	34 (73.9%)	5 (10.9%)	7 (15.2%)	3.2171	2	0.2002
			Polish	27 (57.4%)	6 (12.8%)	14 (29.8%)			
S22.	In many ways, my life is close to ideal	Women	Palestinian	73 (38%)	35 (18.2%)	84 (43.8%)	12.6012	2	0.0018
			Polish	115 (46.7%)	63 (25.6%)	68 (27.6%)			
		Men	Palestinian	22 (47.8%)	7 (15.2%)	17 (37%)	5.2894	2	0.0710
			Polish	14 (29.8%)	16 (34%)	17 (36.2%)			
S23.	My living conditions are perfect	Women	Palestinian	58 (30.2%)	38 (19.8%)	96 (50%)	1.6007	2	0.4492
			Polish	72 (29.3%)	61 (24.8%)	113 (45.9%)			
		Men	Palestinian	13 (28.3%)	7 (15.2%)	26 (56.5%)	0.7063	2	0.7025
			Polish	17 (36.2%)	7 (14.9%)	23 (48.9%)			
S24.	I am satisfied with my life	Women	Palestinian	32 (16.7%)	16 (8.3%)	144 (75%)	10.6195	2	0.0049
			Polish	45 (18.3%)	46 (18.7%)	155 (63%)			
		Men	Palestinian	8 (17.4%)	5 (10.9%)	33 (71.7%)	1.9142	2	0.3840
			Polish	8 (17%)	10 (21.3%)	29 (61.7%)			
S25.	I achieved the most important things in life that I wanted	Women	Palestinian	53 (27.6%)	31 (16.1%)	108 (56.3%)	33.0684	2	<0.0001
			Polish	119 (48.4%)	55 (22.4%)	72 (29.3%)			
		Men	Palestinian	13 (28.3%)	12 (26.1%)	21 (45.7%)	1.6430	2	0.4398
			Polish	19 (40.4%)	9 (19.1%)	19 (40.4%)			
S26.	If I could live my life over again, I would not want to change almost anything	Women	Palestinian	81 (42.2%)	22 (11.5%)	89 (46.4%)	6.1458	2	0.0463
			Polish	121 (49.2%)	39 (15.9%)	86 (35%)			
		Men	Palestinian	19 (41.3%)	7 (15.2%)	20 (43.5%)	0.2649	2	0.8759
			Polish	19 (40.4%)	9 (19.1%)	19 (40.4%)			

Answer: 1 – I do not agree, 2 – I neither agree nor disagree, 3 – I agree.

By performing a chi-squared test, it was shown that the responses of women from Poland and Palestine to statements S21, S22, S24, S25, and S26 statistically significantly depend on the country of origin. However, men’s answers to the above questions do not depend on the nationality of the respondents.

The answers of women and men to statement S23 (“My living conditions are perfect”) do not depend on the respondents’ country of residence. It is worth noting that 25.5% of surveyed women from Palestine and 40.2% from Poland feel lonely, statement S21 (“I feel lonely”). This percentage for men is lower and amounts to 15.2% (Palestine) and 29.8% (Poland). 43.8% of women from Palestine and 27.6% of women from Poland agree with the phrase S22 (“In many ways, my life is close to ideal”). In the case of men from Poland and Palestine, this percentage is approximately 36.5%.

According to the subjective assessment of the respondents, their “living conditions are perfect” (S23) for 50% of women from Palestine and 45.9% from Poland, in the case of men these values are 56.5 and 48.9%, respectively.

Life satisfaction, statement S24 (“I am satisfied with my life”), was indicated by 75% of female students from Palestine and 63% of female students from Poland, as well as 71.7% of male students from Palestine and 61.7% from Poland. In the answers to the question S25 (“I achieved the most important things in life that I wanted”), statistically significant differences were detected in women’s answers. 56.3% of women from Palestine agree with this statement, for women from Poland this percentage is 29.3%. In the case of men, 45.7% of men from Palestine and 40.4% from Poland agreed with this fact.

The same number of Polish men (40.4%) agreed with this statement and disagreed with the phrase S26 (“If I could live my life over again, I would not want to change almost anything”). For Palestinian men, the situation is similar, 43.5% of men agree with this statement and 41.3% disagree. The proportions of responses of women from Poland and Palestine are statistically significantly different. 46.4% of Palestinian women and 35% of Polish women agree with this statement, while 42.2% of Palestinian women and 49.2% of Polish women disagree. The assessment of the effect size for a statistically significant relationship according to Cramer’s *V* is: medium for statement S25, weak for S21, S22, S24, and S26.

## 4 Discussion

Based on the data obtained, it should be noted that after the difficult time of the COVID-19 pandemic, Palestinian students (74.4%) are more satisfied with their lives and Polish students are less satisfied (62.8%). The less optimistic attitude toward life of Polish respondents may result from greater freedom of life than that of Palestinian respondents, who are accustomed to existential difficulties. Psychosocial behaviors examined during the pandemic among students from other populations reveal that COVID-19 generated stressors that were associated with poorer mental health and overall poor well-being. Social stressors, especially relational ones, turned out to be the main problem most strongly associated with mental health indicators (26). The above opinion is echoed by Polish students who reveal their existential eudaimonism to a lesser extent than Palestinian students. Reports from other studies confirm that stress and fears caused by the pandemic have negatively affected the mental and existential well-being of young people (61).

Compared with the results of studies conducted among the Palestinian population during the COVID-19 pandemic it was stated that 70% of youth felt depression and almost 90% felt existential anxiety (10). The observed behaviors may indicate a smooth recovery of the psychosocial condition of Palestinian youth, despite the difficult experiences of the pandemic. Students from Palestine are at the forefront of having an optimistic existential attitude and 54.2% of them believe that they have achieved the most important things they wanted in life (62). The Polish population shares this opinion in 31.1%. Moreover, Palestinian students are more interested in physical fitness and health (69.7%), and Polish students are slightly less interested (61.1%). Both groups of respondents have a similar opinion that their physical form is influenced by healthy food (64.7% of Palestinians, and 63.1% of Polish respondents). This belief is reflected in practicing healthy eating habits. The revealed attitude corresponds to the good physical condition and concern for one’s health declared by both populations. For comparison, other studies conducted among students in the spring of 2020 indicate low physical activity in 51.9% of students and consumption of meals increased by one-third (32.6%), which was mainly caused by boredom (63).

Other studies conducted in the second year of the pandemic indicate that students revealed a higher level of physical activity and a greater demand for this activity, as well as healthier eating habits and better self-assessment of their health (64). It was also noticed that the quality of physical activity has a positive impact on the well-being of students and anti-pandemic attitudes and behaviors (65). Moreover, it was noticed in research conducted in 2022 among students of medical and non-medical faculties, where the latter were more sensitive to changes in health-promoting habits (66). Some researchers emphasize that social support was of great importance during the pandemic, which strengthened the general immunity and gave impetus to search for solutions to eliminate the unfavorable existential situation.

Life satisfaction is influenced by good interpersonal relationships (67). Students of both nationalities declared that they met friends (80% of respondents). However, Polish students (80.9%) devote more time to friends and less time to Palestine (64.3%). Good relationships at home were declared by 85.3% of respondents from Palestine and 73.7% from Poland. The above opinion may confirm the thesis that the COVID-19 pandemic has weakened personal relationships and interactions in the space of friendship. Research conducted on this issue indicates serious disruptions in friendships, both existing ones and building new ones. Weaker mutual social support has also been noticed, which significantly weakens friendship relationships and reduces personal resilience (68). Some researchers suggest disruptions in friendships during (69) the pandemic, especially for first-year college students. The reason for this condition was the imposition of physical distance and home isolation (70). Other studies show that students in friendships felt more aware of the psychological processes involved in maintaining friendships, which in turn made them reflect on the value of friendship in personal development (71).

Scientists’ research suggests that in this space, psychological tools should be developed to generate positive personal interactions during the pandemic (72). Despite frequent meetings with friends declared by both groups of students, many of them revealed a feeling of loneliness. Palestinian respondents reveal this condition more often (66.8%), and Polish respondents feel less lonely (46.4%). Reports from other studies conducted during the COVID-19 pandemic indicate a high level of loneliness, which can be overcome by social support, generating personal immunity and shaping assertiveness (73). Subsequent researchers emphasize the important role of the family as a factor in eliminating the loneliness of young people (46). Scientific reports also show that that during the pandemic the level of loneliness was lower among students staying on campuses and attending stationary lectures. The results highlight the importance of open campuses and in-person lectures for increasing social connections among young people (11). From the above perspective, research results indicate a generally positive assessment of online classes expressed by part-time students. Some studies indicate a beneficial effect of online classes (74), while others negatively evaluate this type of educational tools (34).

Their opinion was dictated by saving material resources and time for commuting to the university, silence at home and the possibility of recording lectures. Respondents also noted the disadvantages, which included: a lack of integration between students and problems with information technology (49). The axiology promoted by young people also results from the religion they profess and influences their happiness and existential satisfaction (75–79). Their opinion was dictated by saving that religious and spiritual values are important for Palestinian and Polish students. Palestinian students (85.3%) are more religiously involved, and Polish students are less committed (26.3%). The above opinion corresponds to the practice of the faith (80, 81).

Among Palestinian research participants, this opinion is shared by 87.8% of respondents, and 21.8% of Polish students consider it an important spiritual factor generating a sense of happiness. To a large extent, the attitude of Palestinian respondents is confirmed by the results of research conducted in October 2021 in Palestine, where over 50% of participants expressed the opinion that religion can be an effective tool in eliminating health problems during COVID-19 (80, 82).

The positive impact of Muslim religious practices eliminates depression during the pandemic, where the greatest effectiveness of this factor was demonstrated by people with a high level of religious beliefs (83). Other longitudinal studies (6 months) showed that the intensity of religious practices increased under the influence of the increasing number of infections and deaths (84). It should be added that the respondents from Palestine (students of the University of Bethlehem) constitute a population where 80% are followers of Islam and 20% of Christianity. Other aspects that influence life satisfaction include factors such as entertainment in youth clubs, traveling, relaxing, listening to music, watching movies, using the phone and spending time. Polish students are more active and involved in all these spheres. Respondents from a Palestinian university revealed that they do not travel as often as Polish ones. The lower percentage of Palestinian respondents’ mobility is due to political difficulties and few of them can get out of their territory. In the context of traveling during the COVID-19 pandemic, researchers have proposed a solution by offering digital travel.

Research results indicate that this form of digital traveling has a positive impact on improving students’ emotional behavior (85). Other studies indicate that students traveling during the pandemic had the opportunity to acquire knowledge about the risks and promote preventive health behaviors while traveling (86). Regarding students’ travel behavior during the pandemic, researchers reveal various observations, pointing out that due to pandemic restrictions, trips to universities were less frequent, but also after the pandemic ended, many students decided to stay at home for fear of contracting the virus (87). Based on the data collected from our research, we can observe the practice of intensive reading, which is similar among both groups of students (54.3% Polish and 51.3% Palestinian).

Other reports from research conducted during the pandemic reveal that reading difficulties were most often associated with stress in students, less so in men who watched TV (44). Certain stimulants for young people most often affect their existential satisfaction. For the surveyed population, smoking and the use of psychoactive substances are not an important aspect, because 90.3 and 92.2% of respondents from Palestine and Poland stated that they do not smoke cigarettes, and 92 and 95.6% do not use psychoactive substances. The practice of using psychoactive substances was confirmed by 5% of surveyed Palestinians and 2.4% of Poles. For comparison, it is worth mentioning the results of research conducted among students during the pandemic, which indicate that 12.58% of respondents declared an increase in the use of psychoactive substances, 70.22% saw no differences, and 17.20% revealed a decrease in the use of stimulants (88). Other research indicates that the pandemic has resulted in increased use of electronic nicotine utilization systems (ENDS), especially by young men. The main factors driving the use of electronic systems were: past use of psychoactive substances, trying conventional cigarettes, and living with other ENDS holders (89).

Possessing things and material resources in general is a factor that affects the sense of satisfaction (90–94). The study confirmed that young Palestinian respondents use help from their parents more often (87.8%), and Polish respondents less (78.2%), because they want to be more independent and engage in paid work more often than Palestinian students (95). Moreover, the unfavorable financial status of Palestinian respondents during the pandemic caused disturbances in mental health (61, 96). This ultimately coincides with overall material satisfaction, which is more favorable among Polish students (77.5%) and less satisfactory among Palestinian students (53.8%). It should be added that the time of the pandemic was more unfavorable in financial terms for Palestinian students who most often work in hotels and catering during their studies because their studies are paid (97). The lack of tourists had a very severe impact on their financial status (98). The situation after the pandemic corresponds to research results which reveal that the financial problems of young people around the world resulted in financial stress, which is a crucial factor affecting mental health and generating family conflicts and depression (99). Eliminating psychosocial problems takes time, and the financial situation of Palestinian respondents may confirm this. Mental well-being is a critical factor in generating overall existential satisfaction. However, greater material security does not entirely translate into mental health, because Palestinian research participants (less wealthy) admit to a lesser extent (23.5%) to unfavorable depressive states than Polish participants (35.8%). Our research shows that more Palestinian respondents are satisfied with their mental state (63%), and fewer Polish respondents (50.9%). The revealed opinions of Palestinian students indicate progress, as studies conducted among Palestinian youth during the pandemic showed a high degree of depression (61). The help in overcoming depression was the observance of epidemiological restrictions and maintaining preventive practices, which could contribute to the psychosocial improvement of students (25). It can therefore be said that the end of the pandemic created a new existential reality, devoid of anxiety and gave the opportunity to generate personal eudaimonism.

The above opinion corresponds to the information obtained from the literature review, which reveals that during and after the COVID-19 pandemic, students experienced increased stress, anxiety and depression caused by isolation and disruptions in learning. The inconvenience subsided as the pandemic waned (56). It should be added that the occurrence of depression and anxiety varies depending on the country and the date of the test. Interestingly, depression was more common among non-Chinese students (100). The factors presented have a different impact on personal life satisfaction in both populations after the pandemic. Despite the more difficult everyday living conditions of young Palestinians, who were less provided with health resources (COVID-19 vaccines), this population believes that their life is in many ways close to ideal (42.9%). Polish students are more reserved in this matter (29%), which translates into lower life satisfaction among Polish respondents. Palestinian research participants on this issue reveal less dissatisfaction. Generally, greater life satisfaction among Palestinian respondents, despite many everyday adversities, may indicate their psychosocial existential potential, which allows them to endure the hardships of life. It should be added that Palestinian youth received vaccines much later than Polish youth. The opinion about life being close to the ideal corresponds to the statement revealed by Palestinian research participants: my living conditions are excellent (51.3%) and to the slightly less optimistic feelings of Polish students (46.4%).

The above opinion is combined with other research that confirms that students’ happiness and satisfaction during the COVID-19 pandemic depend on their psychosocial condition, especially awareness of their strengths, setting goals, decision-making skills and building positive social relationships (81). Other research reports show that a significant factor generating a sense of security and happiness is affiliation with university campuses (11). When expressing their opinions on the factors that determine their sense of happiness and life satisfaction, Palestinian and Polish respondents largely accept their current existential situation and would not like to change anything. This opinion is shared by 45.9% of Palestinian and 35.8% of Polish respondents. This is an optimistic vision of looking at one’s own life, compared to data from the pandemic, where a significant decline in the sense of happiness and life satisfaction among young people was observed, especially due to the illness and death of loved ones and the failure of the health service (72). The presented factors that build the existential sense of satisfaction of Palestinian and Polish students from a social perspective determine what has the greatest impact on life satisfaction and indicate what can be done to strengthen the human condition after the COVID-19 pandemic from a psychosocial perspective in conditions of health threat.

An important factor in this perspective will be the use of creative methods of supporting students to rebuild their mental, social and educational health. The proposed strategies are: access to therapy, which includes individual and group therapy, both online and on-site, so that students can cope with anxiety, depression and other emotional problems, mindfulness and meditation, i.e., organizing regular meditation sessions or mindfulness training, which will help in dealing with stress and improving concentration. Another strategy will be resilience programs, i.e., workshops building mental resilience, which will teach how to cope with adversity and develop adaptive skills. A good tool in creative support for students will be art therapy programs, including artistic workshops in painting, sculpture or photography, which will allow to express emotions and process experiences related to the pandemic. Music therapy and poetry therapy will also be helpful in art therapy. During music sessions, students can create music or take part in group classes, which helps reduce stress and improve mood, and they can also write poetry or prose as a form of expressing emotions and reflection after the difficult time of the pandemic (101).

Mental health campaigns are another vital form of help that can be implemented by organizing webinars, distributing educational materials, or social media campaigns and using mobile applications for meditation, mood tracking, or stress management. Finally, promoting physical activity as a way to improve mental and physical health, by organizing sports classes, group walks or fitness challenges is a way to use creative support methods holistically. Each of these strategies can be adapted to the specific needs of students, and their goal is not only to rebuild mental health after the pandemic but also to support further personal and academic development in the new reality (65).

## 5 Study limitations

It must be acknowledged that this research has some limitations. The first covers the population, which consists only of students. The second is the lack of questions regarding the number of COVID-19 vaccines received and respondents’ feelings after receiving the vaccinations. In this study, it was also possible to ask for opinions on the effectiveness of the received vaccines against COVID-19. Although these data are already included in a previously published article and cover the same population, it seems that combined with questions about life satisfaction, they could answer the question: Does and to what extent does the number of received vaccines against COVID-19 influence satisfaction with life? Based on the data collected in the article, other limitations can be noted that does not fully allow for determining the psychosocial and cultural conditions of the studied populations in terms of life satisfaction after the pandemic, which is undoubtedly an impulse for further research in this matter.

## 6 Conclusion

Research shows that Palestinian students are more satisfied with life after the COVID-19 pandemic and are optimistic about the future. The factors generating this condition are good physical condition, health, good relationships at home, high religious and spiritual involvement and systematic practice of faith. Moreover, Palestinian respondents are less depressed and believe that their life is closer to ideal, even though they feel lonely more often and benefit from financial help from their parents. Polish students see their existential happiness in meeting friends, traveling, relaxing, listening to music, watching movies, and using the phone and good financial situation. Both populations similarly declare that their eudaimonism is influenced by positive interactions with friends, physical activity, healthy food and good eating habits, religious values, and practicing intensive reading. They are not interested in using psychoactive substances and smoking cigarettes. The generally optimistic opinion, which was formed after many negative experiences during the pandemic, is due to many social and health factors, including the use of protective vaccines against COVID-19.

The hypothesis about greater life satisfaction after the pandemic among Palestinian respondents living in health-threatening conditions due to the long-term Israeli-Palestinian conflict was confirmed. Future research directions on life satisfaction and contentment are planned among populations that have suffered the most during the pandemic, for example respondents from Italy and China.

## Data Availability

The original contributions presented in the study are included in the article/supplementary material, further inquiries can be directed to the corresponding author.

## Footnotes

^1^ https://www.ibm.com/in-en
